# Benzo[*b*]tiophen-3-ol derivatives as effective inhibitors of human monoamine oxidase: design, synthesis, and biological activity

**DOI:** 10.1080/14756366.2019.1653864

**Published:** 2019-08-19

**Authors:** Paolo Guglielmi, Daniela Secci, Anél Petzer, Donatella Bagetta, Paola Chimenti, Giulia Rotondi, Claudio Ferrante, Lucia Recinella, Sheila Leone, Stefano Alcaro, Gokhan Zengin, Jacobus P. Petzer, Francesco Ortuso, Simone Carradori

**Affiliations:** a Dipartimento di Chimica e Tecnologie del Farmaco, Sapienza University of Rome, Rome, Italy;; b Pharmaceutical Chemistry, School of Pharmacy, Centre of Excellence for Pharmaceutical Sciences, North-West University, Potchefstroom, South Africa;; c Dipartimento di Scienze della Salute, “Magna Graecia” University of Catanzaro, Campus Universitario “S. Venuta”, Viale Europa Loc. Germaneto, Catanzaro, Italy;; d Net4Science Academic Spin-Off, Campus Universitario “S. Venuta”, Viale Europa Loc. Germaneto, “Magna Graecia” University of Catanzaro, Catanzaro, Italy;; e Department of Pharmacy, “G. d’Annunzio” University of Chieti-Pescara, Chieti, Italy;; f Department of Biology, Science Faculty, Selcuk University, Konya, Turkey

**Keywords:** MAO-B inhibitors, benzothiophene, molecular modelling, rat cortex synaptosomes, antioxidant activity, Parkinson’s disease

## Abstract

A series of benzo[*b*]thiophen-3-ols were synthesised and investigated as potential human monoamine oxidase (hMAO) inhibitors *in vitro* as well as *ex vivo* in rat cortex synaptosomes by means of evaluation of 3,4-dihydroxyphenylacetic acid/dopamine (DOPAC/DA) ratio and lactate dehydrogenase (LDH) activity. Most of these compounds possessed high selectivity for the MAO-B isoform and a discrete antioxidant and chelating potential. Molecular docking studies of all the compounds underscored potential binding site interactions suitable for MAO inhibition activity, and suggested structural requirements to further improve the activity of this scaffold by chemical modification of the aryl substituents. Starting from this heterocyclic nucleus, novel lead compounds for the treatment of neurodegenerative disease could be developed.

## 1. Introduction

Monoamine oxidases (MAOs; EC 1.4.3.4) are mitochondrial bound flavoenzymes, which catalyse the oxidative degradation of amines. The two human isoforms (hMAO-A and B) are quite similar sharing ∼70% sequence identity^1^^,^^2^ and are co-expressed, to almost the same extent, in the majority of human tissues. However, some differences in the distribution exist with hMAO-A being the isoform predominantly expressed in placenta and intestinal tract, while hMAO-B is more abundant in the brain and liver. In the central nervous system (CNS), they modulate monoamine levels and thus participate in the complex system that controls the physiological and functional concentrations of these neurotransmitters. The two MAO enzymes share similar affinity for dopamine, epinephrine, norepinephrine, and tyramine; serotonin is the preferred substrate for hMAO-A, while hMAO-B has high affinity for benzylamine^3^.

The products deriving from hMAO enzymatic activity are aldehydes and the ammonium ion, while the by-product hydrogen peroxide (H_2_O_2_) is formed in order to regenerate the catalytically active form of the FAD cofactor^4^. Although aldehydes do not appear to accumulate in the healthy brain, some studies have shown that elevated concentration of these products may exert cytotoxic effects^5^. Moreover, the abnormal expression or increased activity of hMAOs may lead to the excessive production of H_2_O_2_ that can expose cells to oxidative damage. In this respect, H_2_O_2_ participates in the Fenton reaction^6^ and reacts with certain cations such as Cu^+^ and/or Fe^2+^ which leads to reactive the formation of oxygen species (ROS). ROS are less stable than H_2_O_2_ and immediately react with the surrounding protein systems, which leads to structural/functional damages and cells death^7^. MAO-mediated oxidative stress has been associated with different neurodegenerative pathologies as well as cardiomyopathies. This suggests that reducing the activity of the hMAOs could be useful in order to protect cells^8–10^.

**Figure 1.**
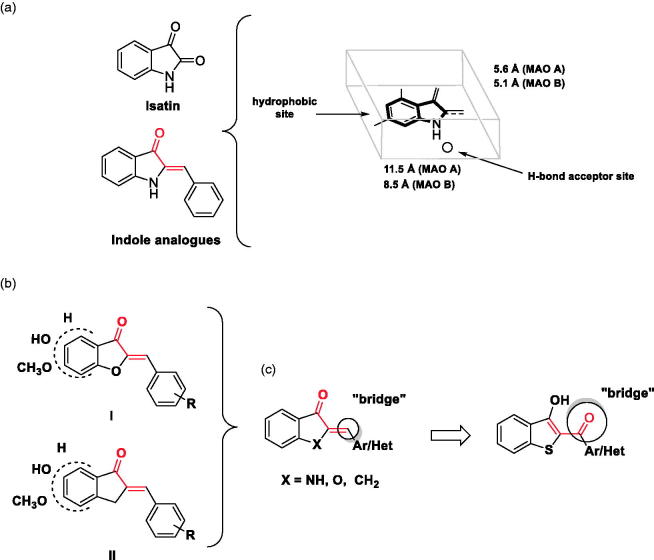
(a) Molecular properties of the pharmacophore for the inhibition of the hMAOs. The part of the structure shown in red corresponds to the chalcone moiety (adapted from reference^13^); (b) Structure of aurone (**I**) and indanone (**II**) derivatives; (c) Benzo[*b*]thiophen-3-ol scaffold: similarities and differences with previously reported compounds.

An extensive number of natural and synthetic compounds have shown effective inhibition of the hMAOs^4^^,^^11^^,^^12^. Keeping in mind the structure and properties of endogenous substrates and reversible inhibitors that bind to hMAOs, Wouters et al.^13^ proposed that ideal MAO inhibitors should be flat molecules (Figure 1(a)) with specific dimensions depending on the isoform (11.5 × 5.5 × 1.8 Å for hMAO-A and 8.5 × 5.1 × 1.8 Å for hMAO-B).

The pharmacophore reported in Figure 1(a) was inspired by isatin and indole analogues which possess different enzyme specificities, inhibiting hMAO-B and hMAO-A, respectively^14^^,^^15^. Although the dimensions of compounds may explain the different affinities for the MAO isoforms (hMAO-A can accommodate larger molecules than hMAO-B), the differences of the electron density in the molecules play an important role in enzyme selectivity. As a matter of the fact, follow-up studies focussed on the isosteric substitution of the nitrogen of the indole system with an oxygen atom or methylene group, to obtain respectively aurone^16^ and indanone derivatives^17^^,^^18^ (Figure 1(b)).

Aurone derivatives (Figure 1(b,I)) showed selective inhibition towards rat MAO-B (rMAO-B) with IC_50_ values ranging from 11.6 to 26.3 µM, without activity against the A isoform. On the other hand, indanone derivatives (Figure 1(b,II)) showed good inhibitory profile in the low micromolar range especially for hMAO-B (0.0052 < IC_50_ hMAO-B (µM) <2.74). All these structures (indole analogues, aurone, and indanone derivatives) share a common structural feature, and is thus similar to the chalcone moiety (highlighted in red), whose ability to bind to hMAO enzymes have been reported in the past by our group^19^. With the aim to explore new structures for the inhibition of hMAO, we proposed a new scaffold based on benzo[*b*]thiophen-3-ol structure (Figure 1(c)). This scaffold retained some similarity with the compounds discussed above, for example the presence of bicyclic system connected with a “bridge” to an aromatic/heteroaromatic ring.

Other important differences among these scaffolds are the isosteric replacement of the oxygen atom of aurones with sulphur and the presence of 1,3-diketonic system that, *via* keto-enol tautomerism, generates the corresponding chalcone while also possessing the potential for metal chelation^20^. This is a very interesting aspect and provides the possibility to obtain multi-target-directed drugs in the light of the evidence that some cations may contribute to neurodegeneration in CNS tissues. As mentioned above, these ions are implicated in the Fenton reaction^6^ which catalyses the production of hydroxyl radicals from hydrogen peroxide, a well-known by-product of MAO enzymatic activity^21^^,^^22^. Since hydroxyl radicals possess a very short half-life, estimated at 1 ns, they can be highly toxic to biomolecules and the use of chelating agents should repair this metal dyshomeostasis leading to reduced damage derived from oxidative stress.

**Scheme 1.**
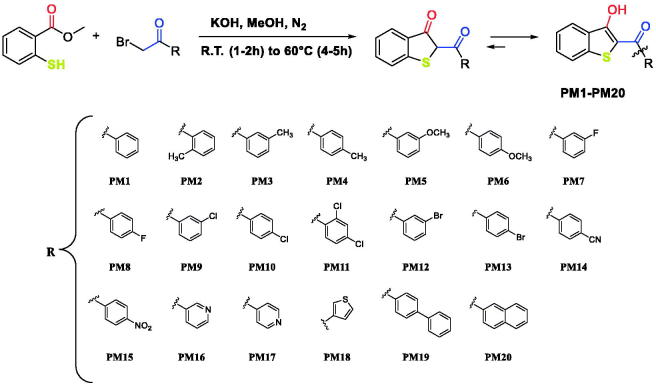
Synthesis of compounds **PM1**-**PM20**.

The structure of benzo[*b*]thiophen-3-ol has been previously studied from a chemical point of view and several research groups have proposed synthetic strategies to obtain this class of compounds^23–25^. Here, we propose a new one-step, very simple synthetic procedure which allowed us to obtain the desired compounds **PM1**-**PM20** (Scheme 1). Methyl 2-mercaptobenzoate and α-bromo acetophenone in equimolar amount, were reacted in methanol in the presence of potassium hydroxide. The reaction was performed in a nitrogen atmosphere (to avoid sulphur oxidation) and at room temperature for 1–2 h. After this time, an excess of potassium hydroxide was added, and the temperature was raised to 60 °C. The completion of reactions was usually reached in 4–5 h producing all the compounds **PM1**-**PM20** in high yields.

**Scheme 2.**
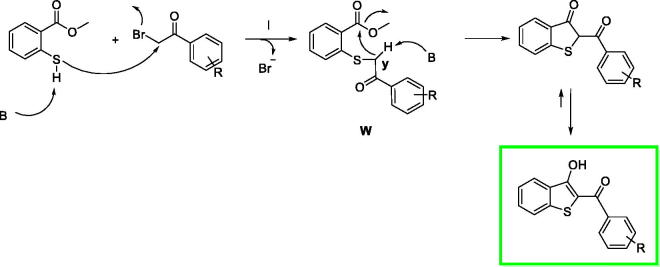
Proposed mechanism of reaction.

We proposed a reaction mechanism for the synthesis of the benzo[*b*]thiophen-3-ol derivatives as reported in Scheme 2.^Scheme 1.^ The first phase of the reaction is the nucleophilic attack of the deprotonated thiol group on α-position of the ketone to obtain the intermediate **W**. After an intramolecular crossed aldolic reaction between the methylene **y** and methyl ester functional group with consequent methanol elimination, the benzo[*b*]thiophen-3-ol is obtained^26^.

The synthesised compounds **PM1**-**PM20** were then evaluated as hMAO inhibitors using the recombinant hMAOs as enzyme sources. The inhibitory activities of compounds **PM1**-**PM20** are summarised in Table 1 along with selectivity index (SI) values given as the ratio (IC_50_ hMAO-A)/(IC_50_ hMAO-B).

**Table 1.** Inhibitory activity (IC_50_) and selectivity index (SI) of compounds **PM1**-**PM20** towards hMAO-A and hMAO-B.

	

a Values are the mean ± SD of triplicate determinations. ^b^Selectivity index for the MAO-B isoform, given as the ratio: (IC_50_ hMAO-A)/(IC_50_ hMAO-B).

Compounds that showed the best IC_50_ values towards hMAO-B (**PM4**, **PM5**, **PM6**, **PM9**, **PM10**, **PM12**, and **PM13**) were also tested in cortex synaptosomes in both basal and LPS-induced inflammatory conditions, to estimate the capability of reducing 3,4-dihydroxyphenylacetic acid/ dopamine (DOPAC/DA) ratio and lactate dehydrogenase (LDH) activity^27–29^. The most active compounds (**PM4**, **PM5**, **PM6**, **PM9**, **PM10**, **PM12**, and **PM13**) were further tested for their antioxidant and metal chelating activity with the aim to demonstrate the possibility of ancillary effects in neurodegenerative disorders. Finally, all the compounds were analysed by molecular modelling to better corroborate the biological data and to further determine which tautomer was responsible for the observed biological activity.

## 2. Experimental protocols

### 2.1. General

Unless otherwise indicated, all reactions were carried out under a positive nitrogen pressure (balloon pressure) in washed and oven-dried glassware. Solvents and reagents were used as supplied without further purification. All melting points were measured on a Stuart^®^ melting point apparatus SMP1 and are uncorrected (temperatures are reported in °C). Fluorescence spectrophotometry was carried out with a Varian Cary Eclipse fluorescence spectrophotometer. ^1^H and ^13^C NMR spectra were recorded at 400 and 101 MHz, respectively, on a Bruker spectrometer using CDCl_3_ and DMSO-d_6_ as the solvents at room temperature. The samples were analysed with a final concentration of ∼30 mg/ml. Chemical shifts are expressed as *δ* units (parts per million) relative to the solvent signal. ^1^H spectra are reported as follows: *δ*_H_ (spectrometer frequency, solvent): chemical shift/ppm (multiplicity, *J*-coupling constant(s), number of protons, assignment). ^13^C spectra are reported as follows: *δ*_C_ (spectrometer frequency, solvent): chemical shift/ppm (*J*-coupling constant C-F, assignment). Multiplicity is abbreviated as follows: br – broad; s – singlet; d – doublet; t – triplet; q – quartette; and m – multiplet. Coupling constants *J* are given in Hertz (Hz). The processing and analyses of the NMR data were carried out with MestreNova. Column chromatography was carried out using Sigma-Aldrich^®^ (St. Louis, MO), silica gel (high purity grade, pore size 60 Å, 230–400 mesh particle size). All the purifications and reactions were monitored by TLC which was performed on 0.2 mm thick silica gel-aluminium backed plates (60 F_254_, Merck, Kenilworth, NJ)Visualisation was carried out under ultra-violet irradiation (254 nm). Elemental analyses for C, H, and N were recorded on a Perkin-Elmer 240 B microanalyzer obtaining analytical results within ± 0.4% of the theoretical values for all compounds. Where given, systematic compound names are those generated by ChemBioDraw Ultra 12.0 following IUPAC conventions. Microsomes from insect cells containing recombinant hMAO-A and hMAO-B (5 mg protein/ml) and kynuramine dihydrobromide were obtained from Sigma-Aldrich (St. Louis, MO).

### 2.2. Chemistry

In an oven dried flask containing a stirring solution of methyl 2-mercaptobenzoate (1.0 equiv.) in methanol (10 ml), freshly ground potassium hydroxide (1.5 equiv.) and the appropriate α-bromoacetophenone (1.0 equiv.) were added. The reaction was performed at room temperature for 1–2 h. After this time, an excess of potassium hydroxide was added (1.5 equiv.) and the temperature was raised to 60 °C. The progression of reaction was monitored by TLC and completion was usually reached in 4–5 h. The reaction was poured into ice-cold water (30 ml) and the pH was adjusted to the value of ∼7 with a 2 N HCl solution to induce the complete precipitation of the desired compound. The resulting benzo[*b*]thiophen-3-ol was collected by vacuum filtration and washed with hot methanol (20 ml). This procedure was used in order to obtain all the compounds **PM1**-**PM20** in high yields and a good level of purity.

#### 2.2.1. (3-Hydroxybenzo[b]thiophen-2-yl)(phenyl)methanone (PM1)

Yellow powder, mp 118–120 °C, 80% yield; ^1^H NMR (400 MHz, DMSO-d_6_): *δ* 7.48–7.52 (m, 1H, benzothiophene), 7.58–7.72 (m, 3H Ar + 1H benzothiophene), 7.92–7.95 (m, 2H, Ar), 7.98 (d, *J*= 8.2 Hz, 1H, benzothiophene), 8.05 (d, *J*= 8.0 Hz, 1H, benzothiophene), 12.02 (brs, 1H, OH, D_2_O exch.). ^13^C NMR (101 MHz, DMSO-d_6_): *δ* 112.2 (benzothiophene), 123.9 (benzothiophene), 124.0 (benzothiophene), 125.5 (benzothiophene), 128.8 (Ar), 129.1 (Ar), 130.4 (benzothiophene), 131.1 (Ar), 133.1 (Ar), 138.6 (benzothiophene), 140.0 (benzothiophene), 160.3 (C_benzothiophene_-OH), 190.9 (C═O). Anal. Calcd for C_15_H_10_O_2_S: C, 70.80; H, 3.96. Found: C, 70.85; H, 3.99.

#### 2.2.2. (3-Hydroxybenzo[b]thiophen-2-yl)(o-tolyl)methanone (PM2)

Yellow powder, mp 131–133 °C, 86% yield; ^1^H NMR (400 MHz, CDCl_3_): *δ* 2.50 (s, 3H, CH_3_), 7.32–7.36 (m, 2H, Ar), 7.43–7.48 (m, 1H Ar + 1H benzothiophene), 7.54–7.58 (m, 1H, benzothiophene), 7.63–7.65 (m, 1H, Ar), 7.71 (d, *J* = 8.2 Hz, 1H, benzothiophene), 8.09 (d, *J*= 8.0 Hz, 1H, benzothiophene), 12.84 (brs, 1H, OH, D_2_O exch.). ^13 ^C NMR (101 MHz, CDCl_3_): *δ* 19.7 (CH_3_), 112.0 (benzothiophene), 123.1 (benzothiophene), 124.0 (benzothiophene), 124.7 (benzothiophene), 125.5 (Ar), 127.4 (Ar), 130.0 (benzothiophene), 130.5 (benzothiophene), 130.9 (Ar), 131.3 (Ar), 136.3 (Ar), 138.3 (Ar), 141.0 (benzothiophene), 160.3 (C_benzothiophene_ -OH), 190.9 (C═O). Anal. Calcd for C_16_H_12_O_2_S: C, 71.62; H, 4.51. Found: C, 71.68; H, 4.52.

#### 2.2.3. (3-Hydroxybenzo[b]thiophen-2-yl)(m-tolyl)methanone (PM3)

Yellow powder, mp 122–124 °C, 76% yield; ^1^H NMR (400 MHz, CDCl_3_): *δ* 2.49 (s, 3H, CH_3_), 7.44–7.48 (m, 2H Ar + 1H benzothiophene), 7.56–7.60 (m, 1H, benzothiophene), 7.76 (d, *J*= 8.2 Hz, 1H, benzothiophene), 7.87–7.89 (m, 2H, Ar), 8.09 (d, *J*= 8.0 Hz, 1H, benzothiophene), 13.49 (brs, 1H, OH, D_2_O exch.). ^13 ^C NMR (101 MHz, CDCl_3_): *δ* 21.5 (CH_3_), 109.7 (benzothiophene), 123.0 (benzothiophene), 124.0 (benzothiophene), 124.7 (benzothiophene), 125.6 (Ar), 128.6 (Ar), 128.9 (Ar), 130.1 (benzothiophene), 130.3 (benzothiophene), 133.4 (Ar), 138.3 (Ar), 138.7 (Ar), 140.8 (benzothiophene), 165.3 (C_benzothiophene_-OH), 192.1 (C═O). Anal. Calcd for C_16_H_12_O_2_S: C, 71.62; H, 4.51. Found: C, 71.64; H, 4.50.

#### 2.2.4. (3-Hydroxybenzo[b]thiophen-2-yl)(p-tolyl)methanone (PM4)

Yellow powder, mp 99–101 °C, 77% yield; ^1^H NMR (400 MHz, CDCl_3_): *δ* 2.49 (s, 3H, CH_3_), 7.35–7.37 (m, 2H, Ar), 7.43–7.47 (m, 1H, benzothiophene), 7.55–7.59 (m, 1H, benzothiophene), 7.75 (d, *J*= 8.2 Hz, 1H, benzothiophene), 7.99–8.01 (m, 2H, Ar), 8.09 (d, *J*= 8.0 Hz, 1H, benzothiophene), 13.6 (brs, 1H, OH, D_2_O exch.). ^13 ^C NMR (101 MHz, CDCl_3_): *δ* 21.7 (CH_3_), 109.5 (benzothiophene), 122.9 (benzothiophene), 123.9 (benzothiophene), 124.7 (benzothiophene), 128.6 (Ar), 129.4 (Ar), 130.0 (benzothiophene), 130.3 (benzothiophene), 135.5 (Ar), 140.7 (benzothiophene), 143.5 (Ar), 165.3 (C_benzothiophene_-OH), 191.4 (C═O). Anal. Calcd for C_16_H_12_O_2_S: C, 71.62; H, 4.51. Found: C, 71.59; H, 4.47.

#### *2.2.5. (3-Hydroxybenzo[b]thiophen-2-yl)(3-methoxyphenyl)methanone* (*PM5)*

Yellow powder, mp 77–79 °C, 83% yield; ^1^H NMR (400 MHz, CDCl_3_): *δ* 3.93 (s, 3H, OCH_3_), 7.16–7.19 (m, 1H, Ar), 7.44–7.49 (m, 1H Ar + 1H benzothiophene), 7.56–7.60 (m, 1H Ar + 1H benzothiophene), 7.66–7.69 (m, 1H, Ar), 7.76 (d, *J*= 8.2 Hz, 1H, benzothiophene), 7.87–7.89 (m, 2H, Ar), 8.09 (d, *J*= 8.1 Hz, 1H, benzothiophene), 13.45 (brs, 1H, OH, D_2_O exch.). ^13^C NMR (101 MHz, CDCl_3_): *δ* 55.5 (OCH_3_), 109.7 (benzothiophene), 119.2 (Ar), 120.8 (Ar), 123.0 (benzothiophene), 124.0 (benzothiophene), 124.8 (benzothiophene), 129.8 (Ar), 130.2 (benzothiophene), 130.3 (benzothiophene), 139.5 (Ar), 140.8 (benzothiophene), 159.8 (Ar), 165.4 (C_benzothiophene_-OH), 191.6 (C═O). Anal. Calcd for C_16_H_12_O_3_S: C, 67.59; H, 4.25. Found: C, 67.61; H, 4.29.

#### 2.2.6. (3-Hydroxybenzo[b]thiophen-2-yl)(4-methoxyphenyl)methanone (PM6)

Yellow powder, mp 181–183 °C, 73% yield; ^1^H NMR (400 MHz, DMSO-d_6_): *δ* 3.88 (s, 3H, OCH_3_), 7.13–7.15 (m, 2H, Ar), 7.49–7.53 (m, 1H, benzothiophene), 7.62–7.66 (m, 1H, benzothiophene), 7.98–8.01 (m, 2H Ar + 1H benzothiophene), 8.03 (d, *J*= 8.0 Hz, 1H, benzothiophene), 12.76 (brs, 1H, OH, D_2_O exch.). ^13^C NMR (101 MHz, DMSO-d_6_): *δ* 56.1 (OCH_3_), 111.4 (benzothiophene), 114.6 (Ar), 123.7 (benzothiophene), 123.9 (benzothiophene), 125.5 (benzothiophene), 130.3 (benzothiophene), 130.7 (benzothiophene), 130.9 (Ar), 131.3 (Ar), 139.7 (benzothiophene), 160.9 (Ar), 163.5 (C_benzothiophene_-OH), 189.5 (C═O). Anal. Calcd for C_16_H_12_O_3_S: C, 67.59; H, 4.25. Found: C, 67.63; H, 4.24.

#### 2.2.7. (3-Fluorophenyl)(3-hydroxybenzo[b]thiophen-2-yl)methanone (PM7)

Yellow powder, mp 133–135 °C, 82% yield; ^1^H NMR (400 MHz, CDCl_3_): *δ* 7.31–7.36 (m, 1H, Ar), 7.45–7.49 (m, 1H, benzothiophene), 7.52–7.62 (m, 1H Ar + 1H benzothiophene), 7.74–7.77 (m, 1H Ar + 1H benzothiophene), 7.88 (d, *J*= 7.7 Hz, 1H, Ar), 8.09 (d, *J*= 8.1 Hz, 1H, benzothiophene), 13.49 (brs, 1H, OH, D_2_O exch.). ^13 ^C NMR (101 MHz, CDCl_3_): *δ* 109.4 (benzothiophene), 115.4 (d, *J*_C-F _= 23.1 Hz, Ar), 119.5 (d, *J*_C-F_ = 21.3 Hz, Ar), 123.0 (benzothiophene), 124.1 (benzothiophene), 124.2 (d, *J*_C-F_ = 3.2 Hz, Ar), 124.9 (benzothiophene), 130.2 (benzothiophene), 130.4 (benzothiophene), 130.4 (d, *J*_C-F _=7.8 Hz, Ar), 140.2 (d, *J*_C-F _=6.8 Hz, Ar), 140.8 (benzothiophene), 162.7 (d, *J*_C-F _=248.5 Hz, Ar), 165.8 (C_benzothiophene_-OH), 190.2 (C═O). Anal. Calcd for C_15_H_9_FO_2_S: C, 66.17; H, 3.33. Found: C, 66.18; H, 3.37.

#### 2.2.8. (4-Fluorophenyl)(3-hydroxybenzo[b]thiophen-2-yl)methanone (PM8)

Yellow powder, mp 127–129 °C, 87% yield; ^1^H NMR (400 MHz, CDCl_3_): *δ* 7.12 (t, *J*= 8.6 Hz, 2H, Ar), 7.34 (t, *J*= 7.6 Hz, 1H, benzothiophene), 7.46 (t, *J*= 7.6 Hz, 1H, benzothiophene), 7.63 (d, *J* = 8.2 Hz, 1H, benzothiophene), 7.95–8.01 (m, 2H Ar + 1H benzothiophene), 13.32 (brs, 1H, OH, D_2_O exch.). ^13^C NMR (101 MHz, CDCl_3_): *δ* 109.2 (benzothiophene), 115.9 (d, *J*_C-F _=21.9 Hz, Ar), 123.0 (benzothiophene), 124.0 (benzothiophene), 124.8 (benzothiophene), 130.2 (benzothiophene), 130.3 (benzothiophene), 131.0 (d, *J*_C-F_ = 9.2 Hz, Ar), 134.4 (d, *J*_C-F _=3.0 Hz, Ar), 140.6 (benzothiophene), 165.4 (d, *J*_C-F _=254.7 Hz, Ar), 165.6 (C_benzothiophene_-OH), 190.2 (C═O). Anal. Calcd for C_15_H_9_FO_2_S: C, 66.17; H, 3.33. Found: C, 66.20; H, 3.39.

#### 2.2.9. (3-Chlorophenyl)(3-hydroxybenzo[b]thiophen-2-yl)methanone (PM9)

Yellow powder, mp 166–168 °C, 89% yield; ^1^H NMR (400 MHz, CDCl_3_): *δ* 7.45–7.52 (m, 1H Ar + 1H benzothiophene), 7.58–7.62 (m, 1H Ar + 1H benzothiophene), 7.76 (d, *J*= 8.2 Hz, 1H, benzothiophene), 7.94–7.96 (m, 1H, Ar), 8.03–8.04 (m, 1H, Ar), 8.09 (d, *J* = 8.0 Hz, 1H, benzothiophene), 13.29 (brs, 1H, OH, D_2_O exch.). ^13^C NMR (101 MHz, CDCl_3_): *δ* 109.4 (benzothiophene), 123.0 (benzothiophene), 124.1 (benzothiophene), 124.9 (benzothiophene), 126.5 (Ar), 128.5 (Ar), 130.0 (Ar), 130.2 (benzothiophene), 130.5 (benzothiophene), 132.6 (Ar), 135.0 (Ar), 139.8 (Ar), 140.8 (benzothiophene), 165.7 (C_benzothiophene_-OH), 190.2 (C═O). Anal. Calcd for C_15_H_9_ClO_2_S: C, 62.40; H, 3.14. Found: C, 62.42; H, 3.11.

#### *2.2.10. (4-Chlorophenyl)(3-hydroxybenzo[b]thiophen-2-yl)methanone* (*PM10)*

Yellow powder, mp 164–166 °C, 88% yield; ^1^H NMR (400 MHz, CDCl_3_): *δ* 7.44–7.48 (m, 1H, benzothiophene), 7.52–7.55 (m, 2H, Ar), 7.57–7.61 (m, 1H, benzothiophene), 7.75 (d, *J*= 8.2 Hz, 1H, benzothiophene), 8.00–8.04 (m, 2H, Ar), 8.08 (d, *J*= 8.1 Hz, 1H, benzothiophene), 13.40 (brs, 1H, OH, D_2_O exch.). ^13 ^C NMR (101 MHz, CDCl_3_): *δ* 109.3 (benzothiophene), 123.0 (benzothiophene), 124.1 (benzothiophene), 124.9 (benzothiophene), 129.1 (Ar), 129.9 (Ar), 130.2 (benzothiophene), 130.4 (benzothiophene), 136.5 (Ar), 139.1 (Ar), 140.7 (benzothiophene), 165.7 (C_benzothiophene_-OH), 190.3 (C═O). Anal. Calcd for C_15_H_9_ClO_2_S: C, 62.40; H, 3.14. Found: C, 62.35; H, 3.17.

#### 2.2.11. (2,4-Dichlorophenyl)(3-hydroxybenzo[b]thiophen-2-yl)methanone (PM11)

White powder, mp 149–151 °C, 88% yield; ^1^H NMR (400 MHz, DMSO-d_6_): *δ* 7.44 (t, *J*= 7.6 Hz, 1H, benzothiophene), 7.58–7.60 (m, 2H Ar + 1H benzothiophene), 7.78 (s, 1H, Ar), 7.94 (d, *J*= 8.1 Hz, 1H, benzothiophene), 8.06 (d, *J*= 8.1 Hz, 1H, benzothiophene), 11.74 (brs, 1H, OH, D_2_O exch.). ^13 ^C NMR (101 MHz, DMSO-d_6_): *δ* 116.5 (benzothiophene), 124.2 (benzothiophene), 125.2 (benzothiophene), 128.0 (Ar), 129.4 (Ar), 130.0 (Ar), 130.1 (benzothiophene), 131.2 (Ar), 132.2 (benzothiophene), 135.1 (Ar), 139.5 (benzothiophene), 140.0 (benzothiophene), 157.3 (C_benzothiophene_-OH), 186.7 (C═O). Anal. Calcd for C_15_H_8_Cl_2_O_2_S: C, 55.75; H, 2.50. Found: C, 55.80; H, 2.53.

#### 2.2.12. (3-Bromophenyl)(3-hydroxybenzo[b]thiophen-2-yl)methanone (PM12)

Yellow powder, mp 169–171 °C, 90% yield; ^1^H NMR (400 MHz, CDCl_3_): *δ* 7.42–7.49 (m, 1H Ar + 1H benzothiophene), 7.58–7.62 (m, 1H, benzothiophene), 7.74–7.77 (m, 1H Ar + 1H benzothiophene), 7.99 (d, *J*= 7.8 Hz, 1H, Ar), 8.09 (d, *J*= 8.0 Hz, 1H, benzothiophene), 8.18–8.19 (m, 1H, Ar), 13.27 (brs, 1H, OH, D_2_O exch.). ^13 ^C NMR (101 MHz, CDCl_3_): *δ* 109.4 (benzothiophene), 123.0 (Ar), 123.1 (benzothiophene), 124.1 (benzothiophene), 124.9 (benzothiophene), 126.9 (Ar), 130.1 (benzothiophene), 130.3 (Ar), 130.5 (benzothiophene), 131.4 (Ar), 135.5 (Ar), 140.0 (Ar), 140.8 (benzothiophene), 165.7 (C_benzothiophene_-OH), 190.1 (C═O). Anal. Calcd for C_15_H_9_BrO_2_S: C, 54.07; H, 2.72. Found: C, 54.10; H, 2.76.

#### 2.2.13. (4-Bromophenyl)(3-hydroxybenzo[b]thiophen-2-yl)methanone (PM13)

Yellow powder, mp 168–170 °C, 84% yield; ^1^H NMR (400 MHz, CDCl_3_): *δ* 7.46 (t, *J*= 7.6 Hz, 1H, benzothiophene), 7.59 (t, *J*= 7.6 Hz, 1H, benzothiophene), 7.70 (d, *J*= 8.5 Hz, 2H, Ar), 7.75 (d, *J*= 8.2 Hz, 1H, benzothiophene), 7.94 (d, *J*= 8.5 Hz, 2H, Ar), 8.08 (d, *J*= 8.0 Hz, 1H, benzothiophene), 13.39 (brs, 1H, OH, D_2_O exch.). ^13 ^C NMR (101 MHz, CDCl_3_): *δ* 109.3 (benzothiophene), 123.0 (benzothiophene), 124.1 (benzothiophene), 124.9 (benzothiophene), 127.7 (Ar), 130.0 Ar), 130.2 (benzothiophene), 130.4 (benzothiophene), 132.0 (Ar), 136.9 (Ar), 140.7 (benzothiophene), 165.7 (C_benzothiophene_-OH), 190.4 (C═O). Anal. Calcd for C_15_H_9_BrO_2_S: C, 54.07; H, 2.72. Found: C, 54.08; H, 2.72.

#### 2.2.14. 4–(3-Hydroxybenzo[b]thiophene-2-carbonyl)benzonitrile (PM14)

Orange powder, mp 176–178 °C, 81% yield; ^1^H NMR (400 MHz, DMSO-d_6_): *δ* 7.48 (t, *J*= 7.6 Hz, 1H, benzothiophene), 7.62 (t, *J*= 7.6 Hz, 1H, benzothiophene), 7.96–8.04 (m, 4H Ar + 1H benzothiophene), 8.08 (d, *J*= 8.1 Hz, 1H, benzothiophene), 11.95 (brs, 1H, OH, D_2_O exch.). ^13^C NMR (101 MHz, DMSO-d_6_): *δ* 114.2 (Ar), 114.5 (benzothiophene), 118.8 (CN), 124.1 (benzothiophene), 124.2 (benzothiophene), 125.4 (benzothiophene), 129.4 (Ar), 130.2 (benzothiophene), 131.8 (benzothiophene), 132.8 (Ar), 140.0 (benzothiophene), 143.1 (Ar), 158.3 (C_benzothiophene_-OH), 188.9 (C═O). Anal. Calcd for C_16_H_9_NO_2_S: C, 68.80; H, 3.25; N, 5.01. Found: C, 68.83; H, 3.29; N, 4.97.

#### 2.2.15. (3-Hydroxybenzo[b]thiophen-2-yl)(4-nitrophenyl)methanone (PM15)

Orange powder, mp 205–207 °C, 79% yield; ^1^H NMR (400 MHz, DMSO-d_6_): *δ* 7.49 (t, *J*= 7.6 Hz, 1H, benzothiophene), 7.58–7.71 (m, 1H, benzothiophene), 7.99 (d, *J*= 8.1 Hz, 1H, benzothiophene), 8.06–8.11 (m, 2H Ar + 1H benzothiophene), 8.38 (d, *J*= 8.1 Hz, 2H, Ar), 11.94 (brs, 1H, OH, D_2_O exch.). ^13^C NMR (101 MHz, DMSO-d_6_): *δ* 114.5 (benzothiophene), 123.9 (Ar), 124.1(benzothiophene), 124.2 (benzothiophene), 125.4 (benzothiophene), 130.1 (Ar), 130.2 (benzothiophene), 131.9 (benzothiophene), 140.0 (benzothiophene), 144.8 (Ar), 149.6 (Ar-NO_2_), 158.1 (C_benzothiophene_-OH), 188.5 (C═O). Anal. Calcd for C_15_H_9_NO_4_S: C, 60.20; H, 3.03; N, 4.68. Found: C, 60.21; H, 3.05; N, 4.69.

#### 2.2.16. (3-Hydroxybenzo[b]thiophen-2-yl)(pyridin-3-yl)methanone (PM16)

Orange powder, mp 120–122 °C, 79% yield; ^1^H NMR (400 MHz, DMSO-d_6_): *δ* 7.47–7.51 (m, 1H, benzothiophene), 7.58–7.64 (m, 1H Pyr + 1H benzothiophene), 7.99 (d, *J*= 8.1 Hz, 1H, benzothiophene), 8.10 (d, *J*= 8.1 Hz, 1H, benzothiophene), 8.21–8.24 (m, 1H, Pyr), 8.79–8.80 (m, 1H, Pyr), 9.00–9.01 (m, 1H, Pyr), 11.92 (brs, 1H, OH, D_2_O exch.). ^13^C NMR (101 MHz, DMSO-d_6_): *δ* 109.5 (benzothiophene), 123.1 (benzothiophene), 123.8 (Pyr), 124.2 (benzothiophene), 125.1 (benzothiophene), 130.0 (benzothiophene), 130.7 (benzothiophene), 136.3 (Pyr), 136.4 (Pyr), 140.8 (benzothiophene), 148.8 (Pyr), 152.4 (Pyr), 165.9 (C_benzothiophene_-OH), 189.3 (C═O). Anal. Calcd for C_14_H_9_NO_2_S: C, 65.87; H, 3.55; N, 5.49. Found: C, 65.89; H, 3.57; N, 5.53.

#### 2.2.17. (3-Hydroxybenzo[b]thiophen-2-yl)(pyridin-4-yl)methanone (PM17)

Orange powder, mp 203–205 °C, 85% yield; ^1^H NMR (400 MHz, CDCl_3_): *δ* 7.46–7.50 (m, 1H, benzothiophene), 7.60–7.64 (m, 1H, benzothiophene), 7.76 (d, *J*= 8.2 Hz, 1H, benzothiophene), 7.85–7.86 (m, 2H, Pyr), 8.10 (d, *J*= 8.1 Hz, 1H, benzothiophene), 8.87–8.89 (m, 2H, Pyr), 13.12 (brs, 1H, OH, D_2_O exch.). ^13^C NMR (101 MHz, CDCl_3_): *δ* 109.4 (benzothiophene), 121.6 (Pyr), 123.1 (benzothiophene), 124.3 (benzothiophene), 125.1 (benzothiophene), 130.0 (benzothiophene), 130.9 (benzothiophene), 141.0 (benzothiophene), 144.7 (Pyr), 150.6 (Pyr), 165.4 (C_benzothiophene_-OH), 189.7 (C═ O). Anal. Calcd for C_14_H_9_NO_2_S: C, 65.87; H, 3.55; N, 5.49. Found: C, 65.85; H, 3.50; N, 5.55.

#### 2.2.18. (3-Hydroxybenzo[b]thiophen-2-yl)(thiophen-3-yl)methanone (PM18)

Yellow powder, mp 182–184 °C, 73% yield; ^1^H NMR (400 MHz, CDCl_3_): *δ* 7.44–7.48 (m, 1H thiophene + 1H benzothiophene), 7.57–7.61 (m, 1H, benzothiophene), 7.78 (d, *J*= 8.2 Hz, 1H, benzothiophene), 7.80–7.81 (m, 1H, thiophene), 8.07 (d, *J*= 8.0 Hz, 1H, benzothiophene), 8.39–8.40 (m, 1H, thiophene), 13.55 (brs, 1H, OH, D_2_O exch.). ^13^C NMR (101 MHz, CDCl_3_): *δ* 109.4 (benzothiophene), 123.0 (benzothiophene), 123.9 (benzothiophene), 124.8 (benzothiophene), 126.6 (thiophene), 127.6 (thiophene), 130.2 (benzothiophene), 130.4 (benzothiophene), 132.2 (thiophene), 140.2 (thiophene), 141.0 (benzothiophene), 165.7 (C_benzothiophene_-OH), 184.6 (C═O). Anal. Calcd for C_13_H_8_O_2_S: C, 59.98; H, 3.10. Found: C, 60.02; H, 3.12.

#### 2.2.19. [1,1'-biphenyl]-4-yl(3-hydroxybenzo[b]thiophen-2-yl)methanone (PM19)

Yellow powder, mp 162–164 °C, 89% yield; ^1^H NMR (400 MHz, CDCl_3_): *δ* 7.44–7.54 (m, 3H Ar + 1H benzothiophene), 7.57–7.61 (m, 1H, benzothiophene), 7.69–7.71 (m, 2H, Ar), 7.77–7.80 (m, 2H Ar + 1H benzothiophene), 8.11 (d, *J*= 8.1 Hz, 1H, benzothiophene), 8.17–8.19 (m, 2H, Ar), 13.61 (brs, 1H, OH, D_2_O exch.). ^13^C NMR (101 MHz, CDCl_3_): *δ* 109.6 (benzothiophene), 123.0 (benzothiophene), 124.0 (benzothiophene), 124.8 (benzothiophene), 127.3 (Ar), 127.4 (Ar), 128.3 (Ar), 129.0 (Ar), 129.1 (Ar), 130.2 (benzothiophene), 130.3 (benzothiophene), 136.9 (Ar), 139.8 (benzothiophene), 140.8 (Ar), 145.5 (Ar), 165.6 (C_benzothiophene_-OH), 191.14 (C═O) Anal. Calcd for C_21_H_14_O_2_S: C, 76.34; H, 4.27. Found: C, 76.38; H, 4.31.

#### 2.2.20. (3-Hydroxybenzo[b]thiophen-2-yl)(naphthalen-2-yl)methanone (PM20)

Yellow powder, mp 126–128 °C, 90% yield; ^1^H NMR (400 MHz, CDCl_3_): *δ* 7.44–7.49 (m, 1H, benzothiophene), 7.57–7.68 (m, 2H Ar + 1H benzothiophene), 7.78 (d, 2H, *J*= 8.2 Hz, 1H, benzothiophene), 7.93–7.95 (m, 1H, Ar), 7.99–8.05 (m, 2H, Ar), 8.09–8.12 (m, 1H Ar + 1H benzothiophene), 8.65 (s, 1H, Ar), 13.58 (brs, 1H, OH, D_2_O exch.). ^13^C NMR (101 MHz, CDCl_3_): *δ* 109.8 (benzothiophene), 123.0 (benzothiophene), 124.0 (benzothiophene), 124.4 (Ar), 124.8 (benzothiophene), 127.0 (Ar), 127.9 (Ar), 128.5 (Ar), 128.7 (Ar), 129.5 (Ar), 129.9 (Ar), 130.2 (benzothiophene), 130.4 (benzothiophene), 132.4 (Ar), 135.3 (Ar), 135.3 (Ar), 139.8 (Ar), 140.8 (benzothiophene), 145.5 (Ar), 165.6 (C_benzothiophene_-OH), 191.14 (C═O). Anal. Calcd for C_19_H_12_O_2_S: C, 74.98; H, 3.97. Found: C, 74.99; H, 4.02.

### 2.3. Biological assays

#### 2.3.1. hMAO-a and hMAO-B inhibition studies

IC_50_ values for the inhibition of hMAO-A and hMAO-B were measured according to the literature protocol^30^^,^^31^ with the commercially available recombinant enzymes (Sigma-Aldrich, St. Louis, MO) serving as enzyme sources. Kynuramine was used as substrate for both hMAO isoforms. The oxidation of kynuramine by the hMAOs yields 4-hydroxyquinoline which was measured by fluorescence spectrophotometry. By assessing the hMAO activities in the presence of a range of different inhibitor concentrations (0.003–100 µM), the IC_50_ values were measured. The enzyme reactions (200 µl) were carried out in white 96-well microtiter plates (Eppendorf, Hamburg, Germany) in potassium phosphate buffer (pH 7.4, 100 mM) and contained kynuramine (50 µM), the test inhibitors spanning at least three order of magnitude (0.003–100 µM) and hMAO-A (0.0075 mg protein/ml) or hMAO-B (0.015 mg protein/ml). The reactions were initiated with the addition of enzyme, incubated for 20 min at 37 °C, and at endpoint were treated with 2 N NaOH (80 µl) to terminate the enzyme reactions. The fluorescence intensity of 4-hydroxyquinoline, the product formed by the MAO-catalysed oxidation of kynuramine, was measured (λ_ex_=310 nm; λ_em_=400 nm). Sigmoidal plots of catalytic rate versus logarithm of inhibitor concentration were constructed and the IC_50_ values were estimated and reported as the mean ± standard deviation (SD) of triplicate measurements.

#### 2.3.2. Rat cortex synaptosomes

Male adult Sprague-Dawley rats (200–250 g) were housed in Plexiglass cages (40 cm × 25 cm × 15 cm), two rats per cage, in climatised colony rooms (22 ± 1 °C; 60% humidity), on a 12 h/12 h light/dark cycle (light phase: 07:00–19:00 h), with free access to tap water and food, 24 h/d throughout the study, with no fasting periods. Rats were fed a standard laboratory diet (3.5% fat, 63% carbohydrate, 14% protein, 19.5% other components without caloric value; 3.20 kcal/g). Housing conditions and experimentation procedures were strictly in accordance with the European Union ethical regulations on the care of animals for scientific research. According to the recognised ethical principles of “Replacement, Refinement and Reduction of Animals in Research”, specimens were obtained as residual material from vehicle-treated rats randomised in our previous experiments approved by Local Ethical Committee (University “G. d’Annunzio” of Chieti-Pescara) and Italian Health Ministry (Project N. 880 definitely approved by Italian Health Ministry on 24th August 2015).

Synaptosomes were prepared from a pool of frontal and parietal cortex, which are more sensitive to oxidative stress compared to other areas such as occipital and dorsal cortex^32^. Briefly, the frontal and parietal cortex was quickly dissected, homogenised in 0.32 M saccharose solution and centrifuged, first at 4000×*g* for 10 min, and then at 12000×*g* for 20 min, to isolate neuronal endings from cell nuclei and glia. The purified synaptosomes were suspended at 37 °C, under O_2_/CO_2_ 95%/5%, pH 7.35–7.45, in Krebs-Ringer buffer (mM: NaCl 125, KCl 3, MgSO_4_ 1.2, CaCl_2_ 1.2, Tris–HCl 10, glucose 10). Then, the synaptosome suspension was divided into fractions (each containing 100 mg of tissue in 3 ml medium) that were incubated at 37 °C, under agitation for 30 min (incubation period), and treated with a pharmacological stimulus as follows: i) Krebs-Ringer buffer (vehicle); ii) vehicle plus oxidant stimulus [LPS 10 μg/ml]; iii) vehicle plus oxidant stimulus and MAO inhibitors (20 nM–1 μM). After the incubation period, synaptosome suspension was centrifuged (12,000×*g* for 20 min) and the supernatant assayed for LDH, DA, and DOPAC determination.

#### 2.3.3. LDH activity determination

LDH activity was measured by evaluating the consumption of NADH in 20 mM HEPES-K^+^ (pH 7.2), 0.05% bovine serum albumin, 20 µM NADH and 2 mM pyruvate using a microplate reader (excitation 340 nm, emission 460 nm) according to manufacturer’s protocol (Sigma-Aldrich, St. Louis, MO). Extracts were tested at 25 µg/ml. Data were obtained from triplicate test and expressed as relative variations compared to vehicle-treated cells^33^.

#### 2.3.4. Neurotransmitter extraction and high-performance liquid chromatography (HPLC) determination

Extracellular DA, 5-HT, and NE levels were analysed through HPLC apparatus consisting of a Jasco (Tokyo, Japan) PU-2080 chromatographic pump and an ESA (Chelmsford, MA) Coulochem III coulometric detector, equipped with microdialysis cell (ESA-5014b) porous graphite working electrode and solid-state palladium reference electrode. The analytical conditions for biogenic amine identification and quantification were selected as previously reported^34^. Briefly, the analytical cell was set at −0.150 V, for detector 1 and at +0.300 V, for detector 2, with a range of 100 nA. The chromatograms were monitored at the analytical detector 2. Integration was performed by Jasco Borwin Chromatography software version 1.5 . The chromatographic separation was performed by isocratic elution on Phenomenex Kinetex reversed-phase column (C18, 150 mm × 4.6 mm i.d., 2.6 µm). The mobile phase was (10:90, *v*/*v*) acetonitrile and 75 mM, pH 3.00 phosphate buffer containing octanesulfonic acid 1.8 mM, EDTA 30 µM and triethylamine 0.015% *v*:*v*. Flow rate was 0.6 ml/min and the samples were manually injected through a 20 µl loop. Neurotransmitter peaks were identified by comparison with the retention time of pure standard. Neurotransmitter concentrations in the samples were calculated by linear regression curve (y = bx + m) obtained with standard. Neither internal nor external standard was necessary for neurotransmitter quantification and all tests performed for method validation yielded results in accordance to limits indicated in official guidelines for applicability in laboratory trials. The standard stock solutions of DA and DOPAC at 2 mg/ml were prepared in bi-distilled water containing 0.004% EDTA and 0.010% sodium bisulphite. The stock solutions were stored at 4 °C. Work solutions (1.25–20.00 ng/ml) were obtained daily progressively diluting stock solutions in mobile phase.

#### 2.3.5. Statistical analysis

GraphPad Prism version 5.01 for Windows (GraphPad Software, San Diego, CA) was used as statistical analysis software. Experiments were performed at least in triplicate and results are presented as mean ± standard deviation (SD). One-way analysis of variance (ANOVA) followed by Newman–Keuls *post-hoc* test was employed to assess significant differences (*p*<.05). As regards the animals randomised for each experimental group, the number was calculated on the basis of the “Resource Equation” N=(E + T)/T^35^.

### 2.4. Molecular modelling

All molecular modelling simulations were carried out by means of the Schrödinger Suite version 2018–1 (Schrödinger LLC, NY)^36^. Maestro GUI^37^ was employed to build the 3D theoretical structures of our derivatives. Both tautomers/conformers were taken into account. Quantum mechanics optimisation was performed by means of Jaguar^38^ software using the DFT B3LYP method and 6–311 G** as basis set. The resulting structure population was estimated according to Boltzmann analysis at 300 K. Protein Data Bank (PDB)^39^ crystallographic structures, deposited with the corresponding codes 2Z5X^40^ and 6FW0^41^, were used as receptor models of hMAO-A and hMAO-B, respectively. Both PDB structures were prepared as follows before the docking simulation: missing atoms and FAD bond order were fixed, hydrogen atoms were added and co-crystallised water molecules and ligands, harmine and chlorophenyl-chromone-carboxamide for 2Z5X and 6FW0, respectively, were removed. After preparation, the Glide^42^ software was used to generate ligand configurations in the enzymatic clefts, which were defined by a regular box of about 64,000 Å^3^ centred on the FAD N5 atom. Ligand structural flexibility was taken into account by the software by a maximum of 10 docking configurations for ligand were generated. The binding affinity was estimated by means of the standard precision (SP) scoring function and the top-ranked complexes, according to Glide Score, were considered for the binding modes analyses. In order to evaluate the non-bonded interaction energy contribution, the Glide Energy and its components Glide E_coul_ and E_vdW_ were examined.

### 2.5. Radical scavenging and chelating activities

#### 2.5.1. Free radical scavenging activity (DPPH)

Each test solution (1 ml) was added to 2,2-diphenyl-1-picrylhydrazyl (DPPH) solution (4 ml, 0.004% methanolic solution). The sample absorbance was noted at 517 nm after 30 min incubation at room temperature in the dark^43^.

#### 2.5.2. 2,2-Azino-bis(3-ethylbenzothiazoline-6-sulfonic acid) (ABTS) radical cation scavenging activity

ABTS^.+^ radical cation was produced *in situ* by reacting a 7 mM ABTS solution with 2.45 mM potassium persulfate and allowing the mixture to incubate for 12–16 h in the dark at room temperature. The ABTS solution was first diluted with methanol to an absorbance of 0.700 ± 0.02 at 734 nm. Each test solution (1 ml) was mixed with ABTS solution (2 ml)^44^.

#### 2.5.3. Evaluation of total antioxidant capacity by phosphomolybdenum assay

Each test solution (0.3 ml) was mixed with 3 ml of reagent solution (0.6 M sulphuric acid, 28 mM sodium phosphate and 4z mM ammonium molybdate). The sample absorbance was read at 695 nm after 90 min incubation at 95 °C^45^.

#### 2.5.4. Cupric ion reducing (CUPRAC) method

Each test solution (0.5 ml) was added to reaction mixture containing CuCl_2_ (1 ml, 10 mM), neocuproine (1 ml, 7.5 mM) and NH_4_Ac buffer (1 ml, 1 M, pH 7.0). Similarly, a blank was prepared as follows: sample solution (0.5 ml) and reaction mixture (3 ml) without CuCl_2_. The absorbances were read at 450 nm after 30 min of incubation at room temperature^46^.

#### 2.5.5. Ferric reducing antioxidant power (FRAP) method

Each sample solution (0.1 ml) was added to the FRAP reagent (2 ml) containing acetate buffer (0.3 M, pH 3.6), 2,4,6-tris(2-pyridyl)-*s*-triazine (TPTZ) (10 mM) in 40 mM HCl and ferric chloride (20 mM) in a ratio of 10:1:1 (*v/v/v*). Then, the absorbance was read at 593 nm after a 30 min incubation at room temperature^47^.

#### 2.5.6. Metal chelating activity on ferrous ions

Each test solution (2 ml) was added to FeCl_2_ solution (0.05 ml, 2 mM). The reaction was initiated by the addition of 5 mM ferrozine (0.2 ml). Similarly, a blank was prepared as follows: test solution (2 ml), FeCl_2_ solution (0.05 ml, 2 mM) and water (0.2 ml). The absorbances of sample and blank were subsequently noted at 562 nm after 10 min incubation at room temperature^48^.

## 3. Result and discussion

### 3.1. In vitro MAO inhibition study

The synthesised compounds **PM1**-**PM20** were evaluated as potential inhibitors of hMAO-A and hMAO-B, and the activities are given as the IC_50_ values in Table 1. Among these derivatives, only compounds **PM2**, **PM17,** and **PM18** were superior inhibitors of hMAO-A compared to hMAO-B, although only to a small extent. All other compounds were selective hMAO-B inhibitors with IC_50_ values in the micromolar/low micromolar range. The simplest compound of this series, **PM1**, containing an unsubstituted phenyl ring bound to the carbonyl “bridge,” showed similar inhibition against both the isoforms with poor selectivity (IC_50_ hMAO-A = 13.3 µM; IC_50_ hMAO-B = 7.39 µM; SI = 1.8). When substitution on the phenyl ring occurred, we observed different effects depending on the position and chemical nature of the substituent. In fact, the presence of a weak electron donor such as the methyl group on phenyl ring, improved inhibition activity towards hMAO-B if placed on the *meta* (**PM3**, IC_50_ hMAO-B = 1.81 µM) or *para* (**PM4**, IC_50_ hMAO-B = 0.47 µM) positions. **PM4** also displayed improved inhibition activity towards hMAO-A (**PM4**, IC_50_ hMAO-A = 2.71 µM), accounting for the slightly reduced SI compared with **PM3** (**PM3**, IC_50_ hMAO-A = 12.6 µM). On the other hand, when this substituent was located at the *ortho*-position, we observed reduced inhibition activity against both the isoforms, with a slight preference for hMAO-A (**PM2**, IC_50_ hMAO-A = 18.7 µM; IC_50_ hMAO-B = 23.4 µM; SI = 0.8). A similar trend was observed for compounds substituted with the methoxy group, which is considered to be a stronger electron donor than the methyl (**PM5** and **PM6**). For these two derivatives, we also recorded different inhibition activities towards hMAO-B, with the best efficacy recorded when the methoxy group was placed on the *para*-position of the phenyl ring (**PM6**, IC_50_ hMAO-B = 0.28 µM). Similar to the methyl-substituted derivatives, the placement of the methoxy substituent on the *meta-*position negatively affected inhibition activity against hMAO-A (**PM5**, IC_50_ hMAO-A = 33.0 µM), thus improving the selectivity index (SI = 42.3). In the light of the above, it may be concluded that electron donor groups improve inhibitory activity towards hMAO-B when they are substituted on the *meta*- and *para*-positions. Furthermore, when these groups are placed on the *meta-*position, they also led to an increase in selectivity due to the reduction of hMAO-A inhibition. A different trend was observed for halogen-substituted derivatives (**PM7**-**PM13**). The data show that when the substituent changed from fluoro to chloro and finally to bromo, there was an increment of inhibitory activity and selectivity towards hMAO-B, according to the increased size and reduced electronegativity of the halogen, with the best inhibition and selectivity shown by compound **PM12** (IC_50_ hMAO-B = 0.35 µM; SI = 180). In the light of the above, we conclude that halogens, which represent electron-withdrawing groups, positively affect both inhibition activity and selectivity when they are placed on the *meta* position (e.g. **PM12**, IC_50_ hMAO-B = 0.35 µM *vs.* **PM13**, IC_50_ hMAO-B = 0.88 µM). This trend differs from that observed with the electron donor groups (e.g. methyl or methoxy). Other compounds such as **PM14** and **PM15**, which are substituted on the *para* position with CN and NO_2_, exhibited hMAO-B inhibition in the low micromolar range (**PM14**, IC_50_ hMAO-B = 4.51 µM; **PM15**, IC_50_ hMAO-B = 2.75 µM) with little selectivity between the two isoforms (**PM14** SI = 4.7; **PM15** SI = 6.8). The presence of a heterocyclic ring for compounds **PM16**-**PM19** negatively affected the inhibition of hMAO-B. Finally, substitution of the phenyl ring with the bulky naphthyl, but not biphenyl, improved both activity and selectivity towards hMAO-B (**PM20**, IC_50_ hMAO-B = 1.08 µM; SI = 39.4).

### 3.2. Evaluation of DOPAC/DA ratio and LDH activity

**Figure 2.**
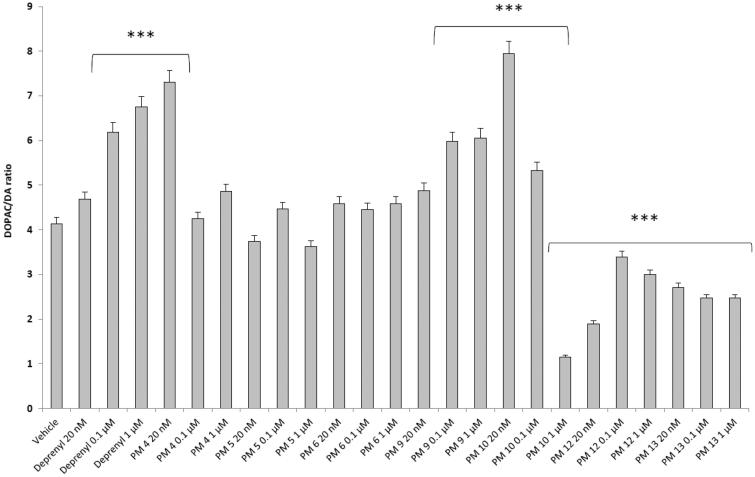
Effect of the **PM** series of inhibitors on DOPAC/DA ratio in rat cortex synaptosomes. ANOVA: *p*<.0001; *post-hoc*: ***p<.001 *vs.* vehicle group. All the compounds and deprenyl were tested at the same concentrations: 20 nM, 0.1 μM, and 1 μM.

**Figure 3.**
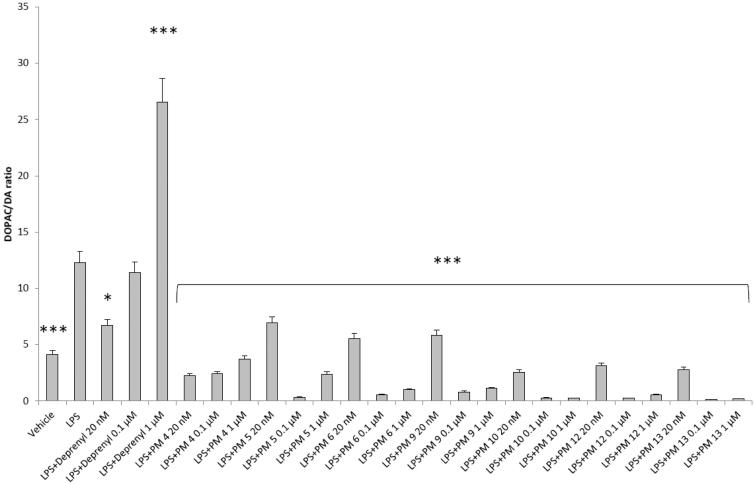
Effect of the **PM** series of inhibitors on DOPAC/DA ratio in rat cortex synaptosomes challenged with LPS. ANOVA: *p*<.0001; *post-hoc*: *p<.05, ***p<.001 *vs.* LPS group. All the compounds and deprenyl were tested at the same concentrations: 20 nM, 0.1 μM and 1 μM.

**Figure 4.**
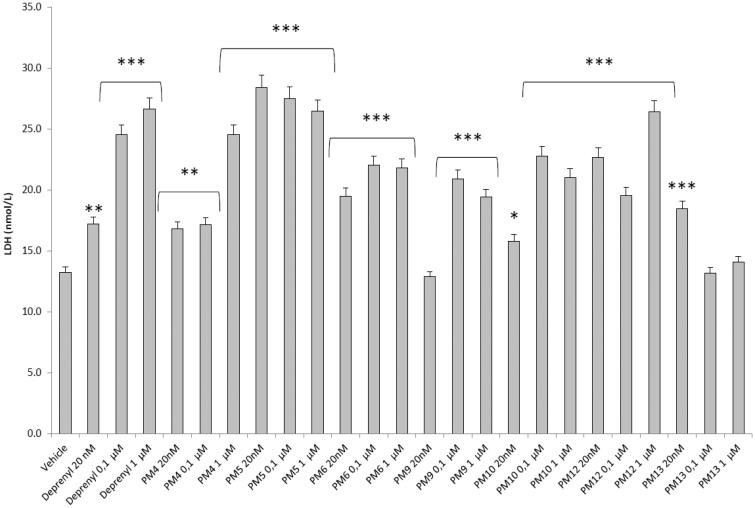
Effect of the **PM** series of inhibitors on LDH activity in rat cortex synaptosomes. ANOVA: *p*<.0001; *post-hoc*: *p<.05, **p<.01, ***p<.001 *vs.* vehicle group. All the compounds and deprenyl were tested at the same concentrations: 20 nM, 0.1 μM, and 1 μM.

**Figure 5.**
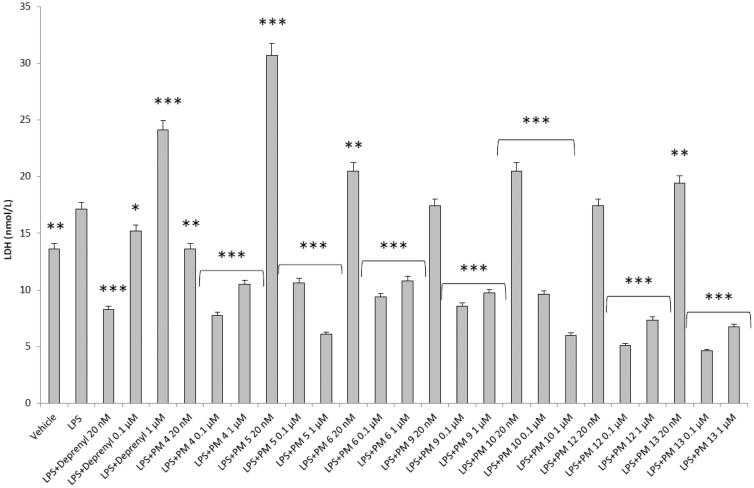
Effect of the **PM** series of inhibitors on LDH activity in rat cortex synaptosomes challenged with LPS. ANOVA: *p*<.0001; *post-hoc*: *p<.05, **p<.01, ***p<.001 *vs.* LPS group. All the compounds and deprenyl were tested at the same concentrations: 20 nM, 0.1 μM, and 1 μM.

Figures 2–5 show that deprenyl, an irreversible and selective MAO-B inhibitor, and the new MAO inhibitors **PM4**, **PM5**, **PM6**, **PM9**, **PM10**, **PM12,** and **PM13** were able to exert modulatory effects on cortex synaptosome DA/DOPAC ratio and LDH activity, in both basal and LPS-induced inflammatory conditions.

Particularly, in Figure 2, it is possible to observe that deprenyl and **PM9** stimulated DOPAC/DA ratio, while **PM10**, **PM12,** and **PM13** inhibited basal DOPAC/DA ratio. On the other hand, no significant effect was exerted by **PM4**, **PM5,** and **PM6**. When the synaptosomes were challenged with an inflammatory LPS stimulus (Figure 3), we observed that deprenyl and all **PM** inhibitors were able to reduce DOPAC/DA ratio. Additionally, the present molecules were more effective than deprenyl, with the highest inhibitory effects displayed by **PM10**, **PM12,** and **PM13**.

DOPAC/DA ratio has long been proposed as an index of MAO-B activity^27^, while microdialysis studies demonstrated the ability of LPS to increase monoamine degradation and extracellular DOPAC levels, in mouse prefrontal cortex^49^. Additionally, we have recently reported that the pro-oxidant stimulus induced by amyloid β-peptide could reduce monoamine levels, in rat cortex synaptosomes^50^^,^^51^. Despite the agreement between the data reported in Figure 3 and the MAO-B inhibitory activity described in Table 1, the contrasting results obtained in basal condition (Figure 2), following **PMs** treatment, suggested the possible onset of pro-inflammatory/pro-oxidant effects, which could have overcome the intrinsic MAO inhibitory activity of these molecules, thus leading to increased DA turnover in rat cortex synaptosomes. In order to test this hypothesis, we performed a second set of experiments to evaluate the effect of deprenyl and the **PM** inhibitors on cortex synaptosome LDH activity, in both basal and LPS-induced inflammatory condition (Figures 4 and 5).

LDH has long been considered a valuable marker of tissue damage^28^^,^^29^. Additionally, antioxidants are able to downregulate their *ex vivo* activity^52^. In this study, we observed that, except for compound **PM13**, most of the **PM** inhibitors upregulated basal LDH activity (Figure 4). Conversely, when synaptosomes were perfused with Krebs-Ringer buffer added with LPS, deprenyl inhibited LDH activity at the lowest concentration (20 nM), which is very close to its MAO-B IC_50_ value (17 nM)^53^, despite exerting a stimulatory effect at the highest tested concentration (1 µM). We cannot exclude that the highest tested deprenyl concentration (1 µM) could be toxic for cortex synaptosomes. Similarly, the **PM** inhibitors displayed a significant LDH inhibitory activity, which is more evident around their respective IC_50_ values, which are included in the range 0.1–1 µM (Table 1). Unlike deprenyl, all the **PM** molecules inhibited LDH activity in the concentration range (0.1–1 μM). Our findings of reduced LPS-induced LDH activity by both deprenyl (20 nM) and **PMs** (0.1–1 μM), in rat cortex synaptosomes, are consistent with the reported antioxidant activity of MAO inhibitors *in vivo*^54^^,^^55^. On the other hand, we should consider that the contrasting finding of stimulation of LDH activity (Figure 4), induced by deprenyl and **PM** inhibitors in basal condition, could be related to the employed *ex vivo* experimental model. Specifically, it is well known that antioxidants in the cell medium could exert pro-oxidative effects, by generating hydrogen peroxide and thus activating adaptive responses of cells to mild oxidative stress. In this context, it is rational to hypothesise that our results of blunted LPS-induced DOPAC/DA ratio and LDH activity, in cortex synaptosomes treated with both deprenyl and **PMs**, could be related to both MAO-B inhibition activity and improved neuron antioxidant defence system. Taken together, these findings support further investigation of the inhibition efficacy of the **PM** series in *in vivo* experimental models of neuroinflammation and oxidative stress. Particularly, future studies should involve inhibitors **PM10**, **PM12,** and **PM13** which exerted the highest inhibitory efficacy on both LPS-induced DOPAC/DA ratio and LDH activity (Figures 3 and 5).

### 3.3. Antioxidant and chelating activity

The most active compounds, investigated in the previous *ex vivo* tests, were also studied as putative antioxidants and metal chelating agents in six *in vitro* spectrophotometric assays (Table 2). In general, they were less potent with respect to the reference compounds (Trolox as antioxidant and EDTA as chelating agent). Only in the phosphomolybdenum test, were **PM4** and **PM5** comparable to the reference drug.

**Table 2.** IC_50_ values in the antioxidant assays (mM).

Samples	DPPH	ABTS	FRAP	CUPRAC	Chelating ability	Phosphomolybdenum assay
PM4	1.13 ± 0.01	>5	3.04 ± 0.03	1.88 ± 0.08	2.63 ± 0.42	4.40 ± 0.07
PM5	1.20 ± 0.02	>5	3.27 ± 0.08	1.14 ± 0.04	1.74 ± 0.19	4.40 ± 0.70
PM6	1.13 ± 0.01	3.18 ± 0.40	3.11 ± 0.11	1.62 ± 0.10	1.89 ± 0.08	>5
PM9	1.31 ± 0.04	2.49 ± 0.30	>5	3.57 ± 0.11	1.97 ± 0.09	>5
PM10	1.10 ± 0.01	3.28 ± 0.44	3.50 ± 0.07	1.41 ± 0.12	2.35 ± 0.13	>5
PM12	1.11 ± 0.01	3.42 ± 0.66	3.95 ± 0.16	1.13 ± 0.06	1.55 ± 0.28	>5
PM13	1.11 ± 0.01	2.68 ± 0.75	2.74 ± 0.10	1.43 ± 0.11	4.71 ± 1.04	>5
Trolox	0.09 ± 0.01	0.15 ± 0.01	0.20 ± 0.01	0.38 ± 0.01	2.63 ± 0.42	2.17 ± 0.10
EDTA	–	–	–	–	0.05 ± 0.01	–

### 3.4. Molecular modelling studies

**Figure 6.**
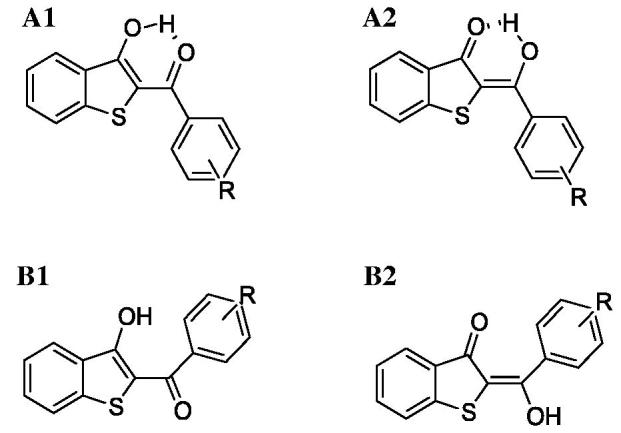
2 D structures of the conformers/tautomers investigated by quantum-mechanics approach for each compound reported in Table 1.

Before evaluating target recognition, the possibility that our compounds could exist in different tautomeric and conformational states have been taken into account. In particular, the keto-enol tautomerism and formation of intramolecular hydrogen bonds (HBs) were investigated. For each compound, four states (Figure 6) were optimised at quantum mechanics level and submitted to the Boltzmann population analysis at 300 K (Table S1).

For all compounds, the **A1** form described a population larger than 95% disregarding the substitution pattern on the aryl ring. Therefore, this form was submitted to molecular docking simulations. Docking analysis was focussed on the binding modes which showed the best theoretical affinity according to the Glide Score. Even if all compounds recognised both hMAO isoforms, they displayed a better theoretical interaction energy towards the hMAO-B, excluding PM11 (Table 3). By computing *r*^2^ between theoretical interaction energies and experimental inhibition data, considered a -log(IC_50_), a strong linear correlation was not found (*r*^2 ^=0.53) but a qualitative accord only. This was not a surprise because IC_50_ does not depend on the ligand target interaction only solvation effect, ligand–-ligand, and ligand–other solutes interaction, not considered in docking simulation, contribute. Analysing the theoretical complexes in terms of interaction energy contribution, we found that, in general, the van der Waals (vdW) component strongly favoured hMAO-B binding compared to hMAO-A (Table 3).

**Table 3.** Theoretical interaction energy and its van der Waals (vdW) and Coulomb (Coul) components are reported (in kcal/mol) for each ligand-target complex.

Compounds	hMAO-A			hMAO-B		
	Interaction Energy			Interaction Energy		
	VdW	Coul	Total	VdW	Coul	Total
PM1	−26.91	−0.81	−27.72	−39.51	0.51	−39.00
PM2	−30.73	−0.24	−30.97	−37.60	−0.79	−38.39
PM3	−15.49	−5.59	−21.08	−45.05	−0.47	−45.52
PM4	−15.12	−4.88	−20.00	−40.88	0.02	−40.87
PM5	−33.95	−1.00	−34.94	−44.92	−0.69	−45.61
PM6	−36.02	−1.23	−37.25	−43.11	0.27	−42.84
PM7	−31.50	−1.77	−33.27	−42.08	0.72	−41.36
PM8	−16.59	−2.93	−19.51	−35.20	−1.44	−36.64
PM9	−16.87	−5.11	−21.97	−46.49	−0.80	−47.28
PM10	−18.41	−3.10	−21.50	−43.35	−0.22	−43.57
PM11	−33.83	−1.49	−35.32	−33.39	−0.40	−33.79
PM12	−19.74	−3.46	−23.20	−47.43	−0.86	−48.29
PM13	−36.96	−2.20	−39.16	−44.01	−0.27	−44.28
PM14	−20.86	−2.78	−23.63	−41.96	−4.55	−46.51
PM15	−37.63	−2.86	−40.49	−43.49	−1.47	−44.96
PM16	−35.75	−1.91	−37.66	−42.30	−0.84	−43.14
PM17	−27.60	−3.00	−30.60	−36.93	−1.22	−38.15
PM18	−38.05	−0.61	−38.66	−41.52	−1.08	−42.59
PM19	−11.88	−0.54	−12.42	−24.22	−0.48	−24.70
PM20	−26.78	−0.69	−27.47	−37.34	−1.16	−38.50

**Figure 7.**
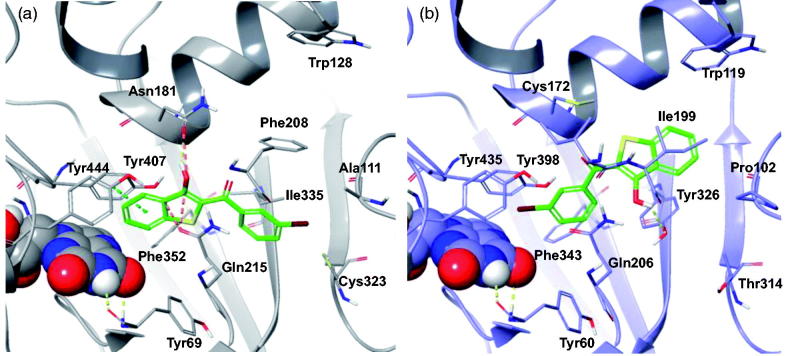
The most stable binding modes of **PM12** in the (a) hMAO-A and (b) hMAO-B active sites, represented in grey and lilac colouring, respectively. The ligand is depicted in polytube with the carbons coloured green, the FAD cofactor is displayed in space fill and the most relevant ligand interacting amino acids are shown as thin tubes. Yellow, light blue, and orange dotted lines represent inter-molecular hydrogen bonds, π–π interactions and unfavourable contacts, respectively.

We ascribe the hMAO-B preference of **PM12**, as reported in Figure 7, to the intermolecular HB with Tyr326 side chain, which is replaced in hMAO-A by the Ile335. Instead, in the hMAO-A pocket, the binding of **PM12** is hampered by the presence of the Phe208 which is replaced by Ile199 in hMAO-B, and thus assumes a different binding mode with the benzothiophene moiety involved in π–π stacking with Tyr407. However, such geometry didn’t prevent unfavourable steric contacts to hMAO-A residues, Asn181 and Gln215.

Regarding the binding modes of the other derivatives, by visual inspections it was observed that, considering the orientation of the benzothiophene portion, they could assume different binding modes in the hMAO-B active site, thus maintaining (**PM2**, **PM4**, **PM6**, **PM7**, **PM8**, **PM11**, **PM13,** and **PM17**) (Figures S1–S8) or losing (**PM1**, **PM3**, **PM5**, **PM9**, **PM10**, **PM14**, **PM15**, **PM16**, **PM18**, **PM19,** and **PM20)** (Figures S9–S19) the intra-molecular HB. In particular, derivatives **PM1**, **PM4**, **PM6**, **PM7**, **PM11**, **PM13,** and **PM19** directed the benzothiophene moiety towards the FAD cofactor forming π–π interactions with active site aromatic amino acids. Among these, only **PM13** does not establish productive contacts with the target. In contrast, the benzothiophene moiety of other compounds was directed towards the entry gorge, which resulted in the positioning of their hydroxyl group close to the side chain of Tyr326 (**PM2**, **PM3**, **PM5**, **PM8**, **PM9**, **PM10**, **PM15**, **PM16**, **PM17**, **PM18**, and **PM20**) or to the Ile199 backbone (**PM20**), thus establishing HB to the target. Thus, the docking results suggested that both π–π interactions and HBs with Tyr326 could play a key role in hMAO-B binding by most of the compounds. Regarding hMAO-A, an intra-molecular HB was present only in the docking poses of compounds **PM1**, **PM17,** and **PM19** (Figures S20–S22). Although the docking complexes of **PM1** and **PM17** were quite similar, **PM19** presented an opposite binding orientation. In this respect, **PM19** fitted in the hMAO-A cleft orientated with the biphenyl moiety towards the outside. In addition, for such compounds conflicts with both tyrosine residues close to the cofactor and with the Ala111 backbone were suggested. Similar to **PM1** and **PM17**, compound **PM11** (Figure S23) bound with the benzothiophene core towards the entry of the active site, but the presence of the 2,4-dichlorine substituted phenyl ring led to the loss of the intra-molecular HB and to steric conflict with Tyr69. This type of unfavourable interactions was present in most of the hMAO-A docking solutions. For example, compounds **PM2**, **PM7**, **PM16,** and **PM18** (Figures S24–S27) displayed similar binding modes, but **PM16** and **PM18** formed HB and π–π interactions with Phe208, whereas for **PM2** and **PM7**, which possessed bulkier substituents, unfavourable contacts with Phe208 and Tyr407 were recorded. These contacts penalised the recognition of derivatives **PM3** (Figure S28) and **PM9** (Figure S29) mainly due to interaction with Tyr407 and Asn181 residues. Thus, the presence of a methyl group or chlorine atom on the *meta*-position of the phenyl ring resulted in similar unfavourable interactions with hMAO-A. For compounds **PM4**, **PM5**, **PM10,** and **PM20** (Figures S30–S33) similar docking poses were predicted. Favourable π–π contacts and very similar recognition of derivatives **PM6**, **PM8**, **PM13**, **PM14**, and **PM15** (Figures S34–S38) by hMAO-A were predicted, although these compounds are differently substituted on the *para*-position of the phenyl ring. Particularly, the poses of **PM6**, **PM13**, and **PM15** were almost identical as well as those of **PM8** and **PM14**. Conversely, for compounds **PM4** and **PM10**, substituted on the *para*-position of the phenyl ring with a methyl group and chlorine atom, respectively, the hydroxyl group was directed towards the cofactor. In conclusion, molecular modelling proposed that benzo[*b*]thiophen-3-ol tautomer **A1** is the most populated state for each compound of our series. In qualitative agreement to the biological assays, all derivatives were able to recognise both hMAOs but preferred hMAO-B in terms of interaction energy and productive contacts. Such a preference seemed to be driven by the vdW interaction energy. Finally, in a large part of the most stable theoretical complexes, the intra-molecular HB was lost and replaced, in hMAO-B only, by a favourable inter-molecular interaction to Tyr326.

## 4. Conclusions

This library of benzothiophen-3-ol derivatives was demonstrated to be a very interesting scaffold to design high potency hMAO-B inhibitors, with IC_50_ values in the low micromolar to nanomolar range. Moreover, the introduction of specific substituents on the phenyl resulted in highly selective inhibition of this isozyme. These compounds were also characterised by an effective *ex vivo* hMAO-B inhibition as well as by limited antioxidant and chelating properties. Furthermore, the binding modes and energies of the designed molecules were predicted *in silico*, thus corroborating the potential of this scaffold for the treatment of neurodegenerative disorders.
